# The impact of delayed adjuvant chemotherapy on survival in gastric cancer patients with and without preoperative chemotherapy

**DOI:** 10.1002/ags3.12911

**Published:** 2025-01-16

**Authors:** Masataka Shimonosono, Takaaki Arigami, Daisuke Matsushita, Yusuke Tsuruda, Ken Sasaki, Kenji Baba, Takao Ohtsuka

**Affiliations:** ^1^ Department of Digestive Surgery Kagoshima University Graduate School of Medical and Dental Sciences Kagoshima Japan

**Keywords:** adjuvant chemotherapy, gastric cancer, neoadjuvant chemotherapy, perioperative medicine, survival

## Abstract

**Aim:**

Adjuvant chemotherapy (AC) is the standard treatment for patients with advanced gastric cancer (GC), yet the optimal timing for its initiation remains unclear. Besides, no studies have definitively established when AC should begin in patients receiving preoperative chemotherapy (PC). This study aimed to determine the optimal timing for initiating AC in patients with GC who underwent curative gastrectomy, either with or without PC.

**Methods:**

A total of 446 patients who underwent curative gastrectomy were evaluated, including 140 who received AC: 72 without PC and 68 with PC. Patients were categorized into two groups based on when they began AC: the early initiation group (within 8 weeks post‐surgery), and the late initiation group (8 weeks or later post‐surgery).

**Results:**

In the non‐PC cohort, the 3‐year relapse‐free survival (RFS) rates were 71% in the early group versus 56% in the late group (*p* = 0.49), while the 3‐year overall survival (OS) rates were 94% versus 73% (*p* = 0.003). Similar trends were observed in the PC cohort; the 3‐year RFS rates were 59% versus 19% (*p* = 0.002), and the 3‐year OS rates were 69% versus 48% (*p* = 0.02). Multivariate analysis identified pretherapeutic distant metastasis (*p* < 0.001) and delayed AC initiation (≥8 weeks) (*p* = 0.001) as independent predictors of worse prognosis.

**Conclusion:**

Delayed initiation of AC is associated with significantly poorer postoperative survival in patients with GC, irrespective of whether PC was administered. These findings emphasize the importance of timely AC initiation to improve long‐term outcomes in this patient population.

## Graphical abstract

Delayed initiation of adjuvant chemotherapy (AC) is associated with significantly poorer postoperative survival in patients with gastric cancer, irrespective of whether preoperative chemotherapy was administered. These findings emphasize the importance of timely AC initiation to improve long‐term outcomes in this patient population.

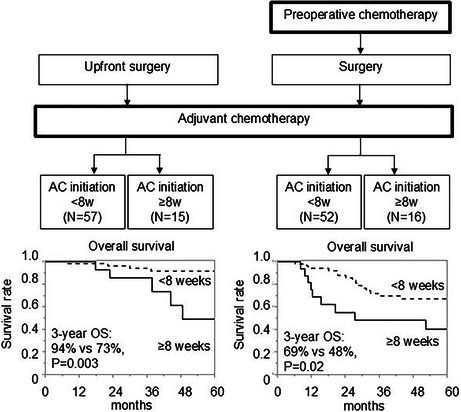

## 1 INTRODUCTION

Gastric cancer (GC) is the sixth most common cancer worldwide and the third leading cause of cancer‐related mortality.^1^ The standard treatment for stage II/III gastric cancer typically involves curative D2 gastrectomy followed by adjuvant chemotherapy (AC), which may include S‐1 monotherapy or more intensive doublet chemotherapy, to reduce the risk of postoperative recurrence.^2^, ^3^, ^4^, ^5^ Recent advances have demonstrated the benefits of neoadjuvant chemotherapy (NAC) in improving postoperative outcomes compared to upfront surgery followed by AC, particularly in Eastern Asia.^6^, ^7^, ^8^ As a result, NAC is now a standard treatment option for clinical T3‐4aN+ or T4b tumors in China^9^ and for tumors with bulky lymph node metastasis in Japan.^10^ Moreover, induction chemotherapy has made conversion surgery feasible for previously unresectable stage IV tumors, although its efficacy remains debated.^11^, ^12^

The clinical benefits of AC in patients undergoing curative resection after preoperative chemotherapy (PC), including NAC and induction chemotherapy, remain unclear. However, the postoperative recurrence rate is high, even after curative resection following PC; therefore, AC is commonly performed as part of perioperative chemotherapy.^7^, ^13^ The optimal timing of AC initiation has not been demonstrated in prospective clinical trials. AC is generally started as soon as possible after surgery to minimize postoperative recurrence; however, practical delays—such as postoperative complications, slow recovery, and decision‐making time—often push back the start date. Recent studies have suggested that delaying AC initiation for 4–8 weeks post‐gastrectomy may negatively impact survival outcomes.^14^, ^15^, ^16^, ^17^ However, no studies have discussed the optimal timing of AC initiation in patients with PC followed by curative gastrectomy.

Therefore, this study aims to fill this gap by retrospectively analyzing the impact of delayed AC initiation on the prognosis of patients with GC who have undergone PC followed by gastrectomy, as well as the patients without PC. This is the first study elucidating the optimal timing of AC initiation both in the patients with or without PC. This research will help to reveal the significance of early AC initiation and its implications for improving survival outcomes in patients with advanced GC.

## 2 PATIENTS AND METHODS

### 2.1 Patients

**FIGURE 1:**
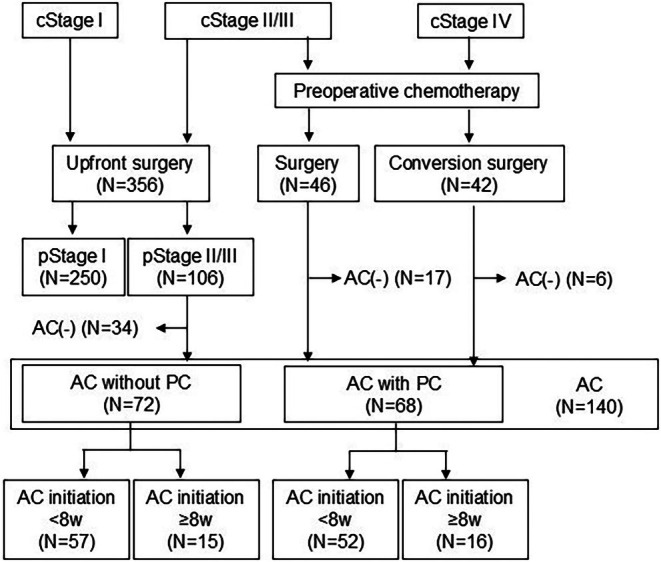
Inclusion criteria and design for this study. Among 446 patients who underwent curative gastrectomy, 140 received adjuvant chemotherapy (AC): 72 after upfront surgery and 68 after surgery with preoperative chemotherapy (PC). Patients were divided into two groups based on the interval between surgery and the initiation of AC: the early AC initiation group (within 8 weeks) and the late AC initiation group (8 weeks or longer).

We retrospectively enrolled 140 patients with GC who underwent curative gastrectomy followed by AC at Kagoshima University between January 2010 and March 2023. This cohort included 72 patients who underwent upfront surgery and were diagnosed with pStage II‐III disease and 68 who received PC followed by surgery. A total of 446 patients underwent curative gastrectomy during this period, only those who proceeded to receive AC were included in the analysis. Patients were divided into two groups based on the interval between surgery and the initiation of AC: the early AC initiation group (within 8 weeks) and the late AC initiation group (8 weeks or longer) (Figure 1). The 8‐week threshold was selected based on retrospective studies indicating that delays beyond this period may negatively affect survival outcomes (14, 16, 17). Ethical approval was obtained from the Institutional Review Board of Kagoshima University (approval number: 240052), and informed consent was provided by all patients. Patient confidentiality was strictly maintained according to ethical guidelines.

### 2.2 Diagnosis and treatment

Clinical and pathological diagnoses were conducted according to the Japanese Classification of Gastric Carcinoma.^18^ Patients with cStage IV GC initially received chemotherapy as per the Japanese treatment guidelines.^10^ If tumors were deemed curatively resectable after chemotherapy, patients proceeded to surgery followed by AC. For resectable cStage II/III tumors, patients underwent curative surgery, with AC administered based on the pathological diagnosis. NAC was considered for patients with Type 3 tumors, bulky N tumors, or Type 4 tumors. The decision to initiate NAC was made following discussions with patients and their families, with informed consent obtained. Postoperatively, patients with pStage II disease typically received S1 monotherapy, while those with pStage III disease were treated with docetaxel and S1. For patients who received PC, the AC regimen was individualized based on the pretherapeutic diagnosis and the tumor's response to chemotherapy. The initiation of AC was canceled or postponed for patients with PS ≥3, hematopoietic disorder, non‐preserved liver or renal functions, active infectious disease, and non‐consent for AC. During or after completion of AC, patients were followed up regularly; observation of clinical symptoms, serum tumor maker tests, and computed tomography (CT) were performed every 3 months up to 2 years post‐surgery and every 6 months up to 3 years post‐surgery. In case that postoperative‐recurrence was suspected, the recurrence was comprehensively diagnosed based on multiple modalities including magnetic resonance imaging, positron emission tomography‐CT, and staging laparoscopy. The median postoperative follow‐up period was 56 months.

### 2.3 Statistical analysis

Survival outcomes, including relapse‐free survival (RFS) and overall survival (OS), were measured from the day of surgery and analyzed using the Kaplan–Meier method. The log‐rank test was employed to compare survival outcomes between the early and late AC initiation groups. Differences in means or proportions between groups were analyzed using the *t*‐test, *χ* ^2^ test, or Fisher's exact test, as appropriate. For multivariate analysis of risk factors contributing to delayed AC initiation, logistic regression analysis was performed with explanatory variables with *p*‐value ≤0.2 on univariate analyses. Cox regression analysis was used to assess prognostic factors for OS, with variables with *p*‐value ≤0.2 on univariate analyses. Statistical analyses were conducted using JMP Pro 16 software (SAS Institute, Cary, NC, USA), with significance set at *p* < 0.05.

## 3 RESULTS

### 3.1 Patient characteristics

In the entire cohort of 140 patients, 109 began AC within 8 weeks of surgery, while 31 started AC 8 weeks or later. In the cohort of 72 patients without PC, 57 started AC within 8 weeks, and 15 started later than 8 weeks (Table 1). In the cohort of 68 patients who received PC, 52 started AC within 8 weeks, and 16 started later than 8 weeks (Table 2). Across both cohorts with or without PC, there were no significant differences in preoperative characteristics between the groups. In the PC cohort, there were no significant differences in the proportion of conversion surgery cases and NAC cases, and the number of PC courses between the groups. The regimens used for PC are listed in Table S1. The incidence of severe postoperative complications was significantly higher in the late AC initiation group compared to the early initiation group in the non‐PC cohort (*p* = 0.008), and a similar trend was shown in the PC cohort (*p* = 0.2). Notably, there were no significant differences between the two groups in terms of pathological diagnosis, the number of AC agents used, or the duration of AC. The regimens used for AC are listed in Table S2. The occurrence of postoperative complications classified as Clavien–Dindo grade ≥3 was identified as an independent risk factor for delayed initiation of AC (*p* = 0.004) (Table 3).

**TABLE 1:** The characteristics of patients who underwent gastrectomy without preoperative chemotherapy.

Factor	AC initiation <8w (*N* = 57)	AC initiation ≥8w (*N* = 15)	*p*‐value
Age (years), median (range)	65 (37–85)	68 (46–88)	0.32
Sex, *n* (%)			
Male	40 (70)	10 (67)	0.76
Female	17 (30)	5 (33)	
Performance status, *n* (%)			
0	51 (89)	13 (87)	0.67
1–2	6 (11)	2 (13)	
Number of comorbidities, *n* (%)			
0–2	54 (95)	15 (100)	1
3–4	3 (5)	0 (0)	
Histological type, *n* (%)			
Differentiated	10 (18)	2 (13)	0.7
Undifferentiated	47 (82)	13 (87)	
Surgical procedure, *n* (%)			
Distal gastrectomy	25 (44)	9 (60)	0.02
Proximal gastrectomy	8 (14)	5 (33)	
Total gastrectomy	24 (42)	1 (7)	
Operative time (min), median (range)	417 (240–731)	433 (185–612)	0.79
Blood loss (mL), median (range)	170 (0–1660)	225 (30–845)	0.78
Post operative complication, *n* (%)			
CD grade 0–2	52 (91)	9 (60)	0.008
CD grade 3–4	5 (9)	6 (40)	
Pathological tumor depth, *n* (%)			
T0	0 (0)	0 (0)	0.47
T1	1 (2)	1 (7)	
T2	7 (12)	1 (7)	
T3	22 (39)	8 (53)	
T4	27 (47)	5 (33)	
Pathological nodal metastasis, *n* (%)			
N0	14 (25)	3 (20)	0.22
N1	17 (30)	1 (7)	
N2	14 (25)	6 (40)	
N3	12 (20)	5 (33)	
Pathological Stage, *n* (%)			
Stage 0	0 (0)	0 (0)	0.24
Stage I	0 (0)	0 (0)	
Stage II	26 (46)	4 (27)	
Stage III	31 (54)	11 (73)	
Adjuvant chemotherapy, *n* (%)			
Single	41 (72)	9 (60)	0.37
Doublet	16 (28)	6 (40)	
Interval to AC (days), median (range)	35 (20–54)	76 (60–179)	<0.001
Duration of AC (months), median (range)	12 (1–48)	12 (2–24)	0.61
Body wight loss (%), median (range)^a^	8.6 (−6.0–27)	9.8 (0–20)	0.6

Abbreviations: AC, adjuvant chemotherapy; CD, Clavien–Dindo.

^a^ Body weight loss from surgery to AC initiation.

**TABLE 2:** The characteristics of patients who received preoperative chemotherapy.

Factor	AC initiation <8w (*N* = 52)	AC initiation ≥8w (*N* = 16)	*p*‐value
Age (years), median (range)	66 (33–88)	65 (52–76)	0.92
Sex, *n* (%)			
Male	40 (77)	12 (75)	0.88
Female	12 (23)	4 (25)	
Performance status, *n* (%)			
0	44 (85)	13 (81)	0.75
1–2	8 (15)	3 (19)	
Number of comorbidities, *n* (%)			
0–2	47 (90)	14 (88)	0.66
3–4	5 (10)	2 (12)	
Histological type, *n* (%)			
Differentiated	19 (37)	7 (44)	0.6
Undifferentiated	33 (63)	9 (56)	
Pretherapeutic distant metastasis, *n* (%)			
M0	26 (50)	6 (38)	0.38
M1	26 (50)	10 (62)	
Preoperative chemotherapy, *n* (%)			
Conversion surgery	26 (50)	10 (62)	0.38
Neoadjuvant chemotherapy	26 (50)	6 (38)	
Number of courses of PC, median (range)	5 (2–23)	4 (1–32)	0.38
Surgical procedure, *n* (%)			
Distal gastrectomy	22 (42)	6 (38)	0.61
Proximal gastrectomy	7 (14)	1 (6)	
Total gastrectomy	23 (44)	9 (56)	
Operative time (min), median (range)	456 (224–649)	483 (348–853)	0.05
Blood loss (mL), median (range)	550 (0–2440)	775 (6–2855)	0.2
Post operative complication, *n* (%)			
CD grade 0–2	47 (90)	12 (75)	0.2
CD grade 3–4	5 (10)	4 (25)	
Pathological tumor depth, *n* (%)			
T0	5 (10)	2 (12)	0.58
T1	3 (6)	0 (0)	
T2	5 (10)	3 (19)	
T3	22 (42)	8 (50)	
T4	17 (32)	3 (19)	
Pathological nodal metastasis, *n* (%)			
N0	25 (48)	6 (37)	0.89
N1	7 (14)	3 (19)	
N2	11 (21)	4 (25)	
N3	9 (17)	3 (19)	
Pathological stage, *n* (%)			
Stage 0	5 (10)	2 (12)	0.18
Stage I	4 (8)	3 (19)	
Stage II	25 (48)	3 (19)	
Stage III	18 (34)	8 (50)	
Adjuvant chemotherapy, *n* (%)			
Single	27 (52)	8 (50)	0.89
Doublet	25 (48)	8 (50)	
Interval to AC (days), median (range)	34 (13–55)	73 (56–126)	<0.001
Duration of AC (months), median (range)	12 (2–60)	8 (1–30)	0.24
Body wight loss (%), median (range)^a^	8.3 (−5.5–21)	9.4 (−11–18)	0.95

Abbreviations: AC, adjuvant chemotherapy; CD, Clavien–Dindo; PC, preoperative chemotherapy.

^a^ Body weight loss from surgery to AC initiation.

**TABLE 3:** Univariate and multivariate analyses for delayed adjuvant chemotherapy.

	Univariate analysis		Multivariate analysis	
	Odds ratio (95% CI)	*p*‐value	Odds ratio (95% CI)	*p*‐value
Age				
≥75	1.76 (0.68–4.55)	0.24	‐	‐
Sex				
Male	0.87 (0.37–2.15)	0.79	‐	‐
Performance status				
PS1‐2	1.30 (0.43–3.96)	0.64	‐	‐
Number of comorbidities				
≥3	0.87 (0.13–3.71)	0.86	‐	‐
Pretherapeutic distant metastasis				
M1	1.44 (0.61–3.45)	0.41	‐	‐
Preoperative chemotherapy				
Yes	1.17 (0.53–2.60)	0.7	‐	‐
Surgical procedure				
Total gastrectomy	0.63 (0.27–1.46)	0.28	‐	‐
Operative time				
≥400 min	1.91 (0.75–4.83)	0.17	1.55 (0.60–4.34)	0.37
Blood loss				
≥200 min	1.37 (0.59–3.19)	0.47	‐	‐
Postoperative complication				
≥CD grade 3	4.71 (1.73–12.9)	0.003	4.67 (1.66–13.4)	0.004
Pathological tumor depth				
≥pT3	0.69 (0.27–1.75)	0.43	‐	‐
Pathological nodal metastasis				
pN+	1.15 (0.51–2.59)	0.73	‐	‐
Pathological stage				
pStage III	1.87 (0.84–4.32)	0.13	2.04 (0.87–5.00)	0.1
Body weight loss^a^				
≥10%	1.37 (0.60–3.06)	0.13	1.15 (0.48–2.70)	0.75

Abbreviations: CD, Clavien–Dindo; CI, confidence interval; PS, performance status.

^a^ Body wight loss from surgery to AC initiation.

### 3.2 Survival outcome

**FIGURE 2:**
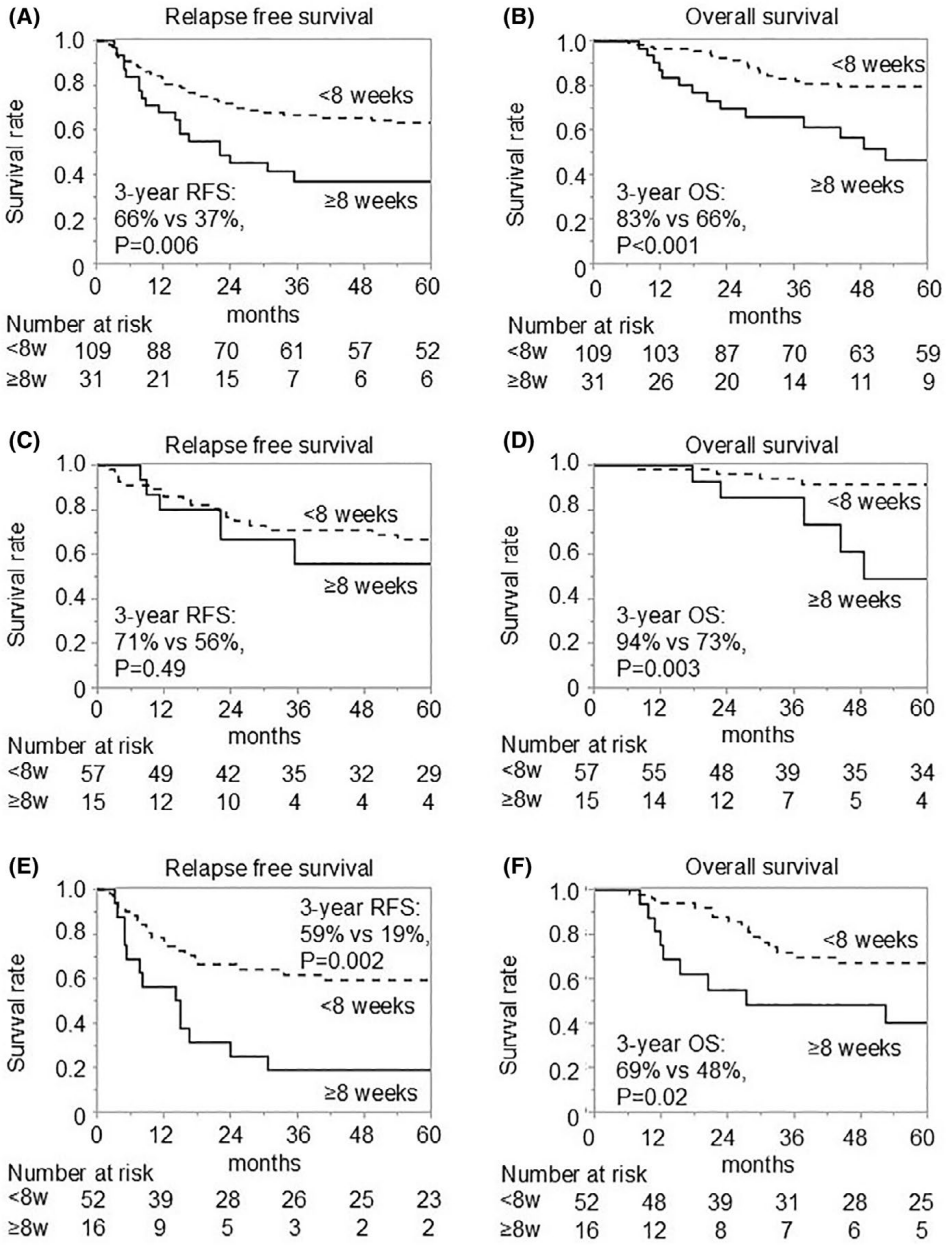
Postoperative survival outcomes. Kaplan–Meier curves for relapse‐free survival (RFS) (A) and overall survival (OS) (B) in the entire patient cohort are shown, as well as RFS (C) and OS (D) in the cohort without preoperative chemotherapy (PC), and RFS (E) and OS (F) in the cohort with PC. Adjuvant chemotherapy (AC) was initiated within 8 weeks after surgery in the <8 weeks group, and at 8 weeks or later in the ≥8 weeks group.

The early AC initiation group demonstrated significantly better RFS compared to the late AC initiation group, both in the entire cohort (3‐year RFS: 66% vs. 37%, *p* = 0.006) (Figure 2A) and in the PC cohort (3‐year RFS: 59% vs. 19%, *p* = 0.002) (Figure 2E), while RFS tended to be higher in the early AC initiation group than in the late AC initiation group in the non‐PC cohort (3‐year RFS: 71% vs. 56%, *p* = 0.49) (Figure 2C). Similarly, OS was notably higher in the early AC initiation group than in the late initiation group in the entire cohort (3‐year OS: 83% vs. 66%, *p* < 0.001) (Figure 2B), as well as in the non‐PC cohort (3‐year OS: 94% vs. 73%, *p* = 0.003) (Figure 2D) and in the PC cohort (3‐year OS: 69% vs. 48%, *p* = 0.02) (Figure 2F).

**FIGURE 3:**
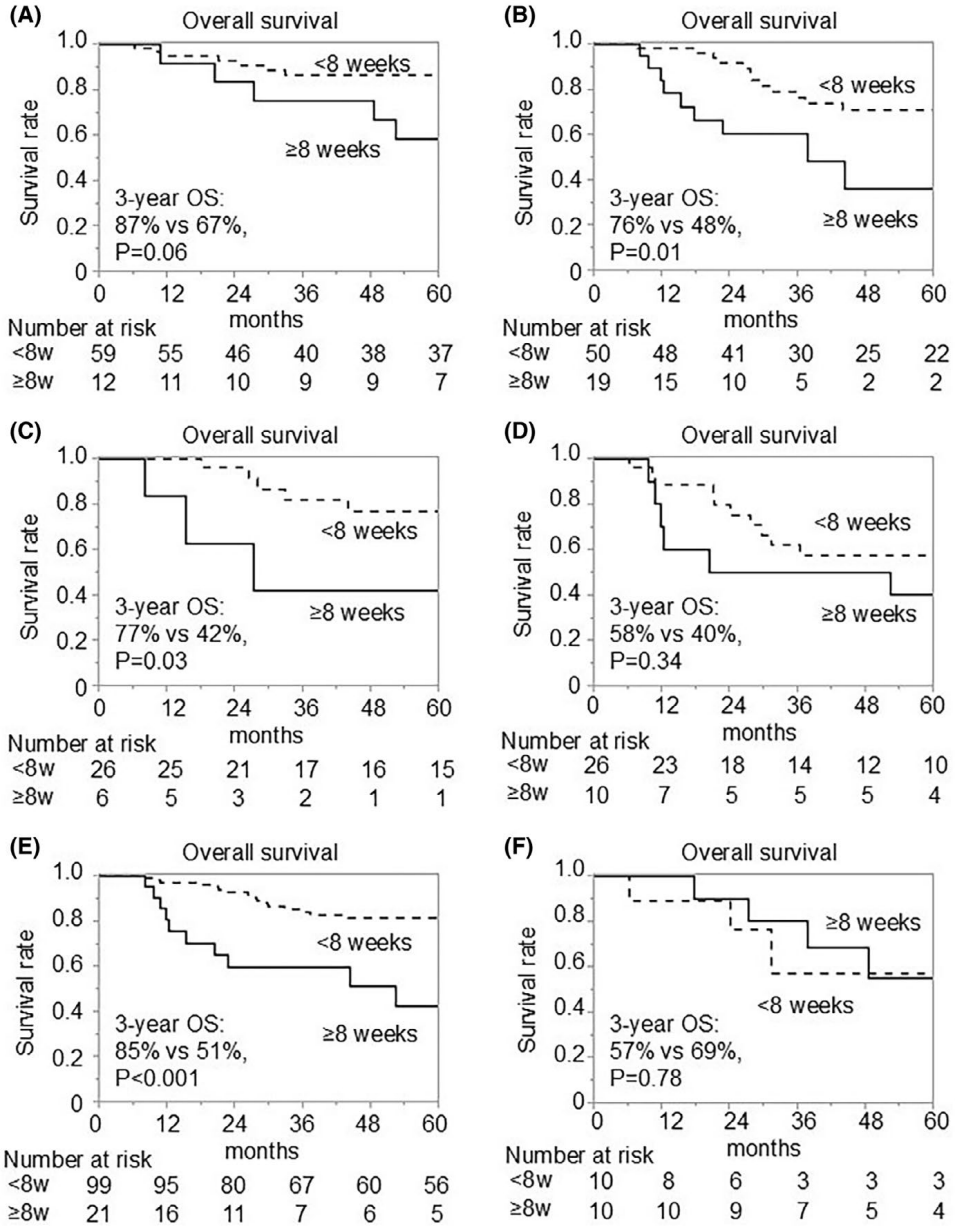
Sub‐group analyses for postoperative survival outcomes. Kaplan–Meier curves for overall survival (OS) in the pStage I‐II group (A) and pStage III group (B) are shown, as well as OS in the neoadjuvant chemotherapy group (C) and the conversion surgery group (D), and OS in the Clavien–Dindo grade <3 group (E) and the Clavien–Dindo grade ≥3 group (F). Adjuvant chemotherapy (AC) was initiated within 8 weeks after surgery in the <8 weeks group, and at 8 weeks or later in the ≥8 weeks group.

Sub‐group analyses showed that OS tended to be higher in the early AC initiation group than in the late AC initiation group in stage I/II cohort (3‐year OS: 87% vs. 67%, *p* = 0.06) (Figure 3A), and OS was significantly higher in the early AC initiation group than in the late AC initiation group in the stage III cohort (3‐year OS: 76% vs. 48%, *p* = 0.01) (Figure 3B). OS was significantly higher in the early AC initiation group than in the late AC initiation group in the NAC cohort (3‐year OS: 77% vs. 42%, *p* = 0.03) (Figure 3C), while there was no difference in OS in the conversion surgery cohort between the early AC initiation group and the late AC initiation group (3‐year OS: 58% vs. 40%, *p* = 0.34) (Figure 3D). On the other hand, OS was significantly higher in the early AC initiation group than in the late AC initiation group in the Clavien–Dindo grade <3 cohort (3‐year OS: 85% vs. 51%, *p* < 0.001) (Figure 3E), however there was no significant difference in OS between the early and late AC initiation groups in the Clavien–Dindo grade ≥3 cohort (3‐year OS: 57% vs. 69%, *p* = 0.78) (Figure 3F).

### 3.3 Multivariate analysis

Multivariate analysis identified pretherapeutic distant metastasis (*p* < 0.001) and delayed AC initiation (≥8 weeks) (*p* = 0.001) as independent predictors of worse prognosis (Table 4). These findings suggest that both preoperative tumor characteristics and postoperative treatment timing critically influence patient prognosis.

**TABLE 4:** Univariate and multivariate analyses for OS.

	Univariate analysis		Multivariate analysis	
	HR (95% CI)	*p*‐value	HR (95% CI)	*p*‐value
Age				
≥75	0.89 (0.34–2.30)	0.81	‐	‐
Sex				
Male	0.53 (0.27–1.07)	0.08	0.47 (0.22–1.00)	0.05
Performance status				
PS1‐2	1.41 (0.58–3.40)	0.45	‐	‐
Pretherapeutic distant metastasis				
M1	3.06 (1.56–6.00)	0.001	5.48 (2.35–12.8)	<0.001
Histological type				
Undifferentiated	2.17 (0.84–5.60)	0.11	2.69 (0.92–7.83)	0.07
Surgical procedure				
Total gastrectomy	1.56 (0.80–3.06)	0.2	1.06 (0.46–2.43)	0.46
Postoperative complication				
≥CD grade 3	1.72 (0.75–3.95)	0.2	1.88 (0.71–4.96)	0.2
Pathological tumor depth				
≥pT3	2.26 (0.80–6.43)	0.13	1.75 (0.37–8.28)	0.48
Pathological nodal metastasis				
pN+	0.72 (0.34–1.50)	0.38	‐	‐
Pathological stage				
pStage III	1.95 (0.98–3.92)	0.06	4.05 (0.51–32.2)	0.19
Initiation of AC				
≥8w	3.09 (1.56–6.12)	0.001	3.33 (1.60–6.90)	0.001
Body weight loss^a^				
≥10%	0.62 (0.30–1.28)	0.2	0.52 (0.23–1.15)	0.11

Abbreviations: AC, adjuvant chemotherapy; CD, Clavien–Dindo; CI, confidence interval; HR, hazard ratio; PS, performance status.

^a^ Body wight loss from surgery to AC initiation.

## 4 DISCUSSION

This study demonstrated that delayed AC initiation beyond 8 weeks was associated with worse postoperative survival in patients who had undergone curative gastrectomy, regardless of the presence of PC. The purpose of AC is to minimize the potential risk for recurrence by eradicating the residual tumor cells or circulating tumor cells. A previous study demonstrated that primary tumor resection in a mouse model accelerated the growth of residual tumor cells via the conversion of non‐cycling cells in the G0 phase into proliferation.^19^ The kinetics of cell proliferation in vivo have shown that the cells grow rapidly at first and slow down gradually.^20^ The effect of most cytotoxic agents depends on the status of proliferation and cell cycle, therefore early AC initiation is theoretically expected to be effective.

Several retrospective studies have similarly reported that a longer interval between surgery and AC was linked to poorer prognosis, with cutoffs ranging from 4 to 8 weeks.^14^, ^15^, ^16^, ^17^ For instance, Park et al. found that patients who initiated AC more than 8 weeks post‐surgery had shorter OS compared to those who started within 8 weeks (5‐year OS: 51.7% vs. 62.8%, *p* = 0.037).^16^ Similarly, Huang et al. reported that in a propensity score‐matched cohort of patients with stage II/III GC, 5‐year RFS rates were 57.6% for those who initiated AC within 8 weeks versus 46.4% for those who began AC after 8 weeks (*p* = 0.028).^14^ Conversely, Greenleaf et al. and Fujitani et al. found no significant impact of AC initiation timing on survival.^21^, ^22^ A large meta‐analysis of 34 comparative studies, encompassing 141 853 patients, revealed that delaying AC initiation beyond 6–8 weeks was associated with inferior OS (HR = 1.2, 95%CI 1.04–1.38; *p* = 0.01).^17^ While prior studies have highlighted the benefits of early AC initiation in patients with stage II/III GC undergoing upfront surgery followed by AC, none have specifically addressed the optimal timing of AC initiation for patients with PC. The efficacy of AC in patients with PC remains controversial. Several retrospective studies have reported survival benefits of AC in perioperative chemotherapy settings.^23^, ^24^, ^25^ For instance, Van Putten et al. showed in a propensity score‐matched analysis that patients receiving PC with AC had better OS compared to those receiving PC alone (HR = 0.84, 95% CI 0.71–0.99).^24^ Rahman et al. also found that patients who received both PC and AC tended to be younger, had lower performance status grades, and experienced fewer surgical complications compared to those receiving PC only. In their study, after weighting, the median OS was 62.7 months for the PC + AC group compared to 50.4 months for the PC‐only group (HR = 0.84, 95%CI 0.77–0.94).^25^ Conversely, Drake et al. reported no significant difference in survival between the two groups in a propensity score‐matched analysis, with a median OS of 56.8 months for PC + AC and 52.5 months for PC only (*p* = 0.131).^26^ Their study suggested that patients who did not receive AC were generally older and had higher comorbidity scores than those who received AC. Given the heterogeneity in postoperative physical conditions among patients undergoing gastrectomy, it is plausible that patients with worse physical status were less likely to receive AC. Thus, evaluating AC efficacy retrospectively introduces challenges, and findings should be interpreted with caution. In the present study, there were no significant differences in patient characteristics between the early and late AC initiation groups, aside from the incidence of postoperative complications (Tables 1 and 2). These data suggest that AC should be initiated within 8 weeks, and they may also imply a potential benefit of AC even in patients with PC who underwent gastrectomy.

In the present study, the results of RFS in the non‐PC cohort and the PC cohort suggested that delaying AC initiation has a stronger impact on survival in the PC cohort than in the non‐PC cohort (Figure 2C,E). Sub‐group analysis showed that early AC initiation significantly improved OS in the stage III cohort, but the effect was limited in the stage I‐II cohort (Figure 3A,B). Park et al. also reported that the early AC initiation (<4 weeks post‐surgery) improved OS in patients with stage III (5‐year OS: 85.6% vs. 47.7%, *p* = 0.008), but not in patients with stage II.^16^ These findings may suggest that the early initiation of AC is vital to improve postoperative survival in patients with more advanced tumors, such as patients with stage III or patients requiring PC. On the other hand, delayed initiation of AC significantly worsened OS in the NAC cohort, but not in the conversion surgery cohort (Figure 3C,D). One reason for these results may be that the potential risk for recurrence was high in the conversion surgery cohort, and the power of AC was insufficient. In many cases, a single agent was used as AC following conversion surgery in this study. We may need to use more potent agents or combination therapy including molecular‐targeted agents to improve the survival outcome in this cohort. Additionally, the early initiation of AC can be less necessary in the conversion surgery cohort, as patients have been treated with PC for longer periods in comparison with that in the NAC cohort.

Several studies have identified factors contributing to delayed AC initiation, such as older age, higher comorbidity burden, and the occurrence of postoperative complications.^14^, ^16^, ^21^, ^22^ In this study, the incidence of postoperative complications was the sole independent predictor of delayed AC initiation (Table 3). This contrasts with findings from a recent meta‐analysis of observational studies, which identified postoperative complications as a negative prognostic factor for patients with GC undergoing gastrectomy.^27^ Our results indicated that while delayed AC initiation was an independent predictor of poorer prognosis, the incidence of postoperative complications was not (Table 4). We conducted a sub‐group analysis according to the presence of postoperative complication (Clavien–Dindo grade ≥3) to reveal the relationship between the timing of AC initiation and postoperative complication. The delayed AC initiation significantly worsened OS in patients without postoperative complications as expected (Figure 3E). However, notably, OS was similar regardless of the timing of AC initiation in patients with postoperative complications (Figure 3F). These results may suggest that the occurrence of postoperative complications attenuates the effect of AC. Postoperative complication induces local and/or systemic inflammation, and inflammatory cytokines can suppress cytotoxic T‐cell immunity and activate anti‐apoptotic programs, leading to chemo‐resistance.^28^ Separately, the use of antibiotics during the treatment of postoperative complications can disrupt the gut microbiota, resulting in the suppression of tumor immunity.^29^ Among altered gut microbiota, *F. nucleatum* is known as a causal species of chemo‐resistance via activation of the autophagy pathway.^30^ These findings underscore the importance of preventing postoperative complications, which could facilitate timely AC initiation, maintain the intact chemotherapeutic effect, and in turn, improve survival outcomes for patients with GC following gastrectomy.

This study has several limitations. First, it was a single‐institution retrospective study with a relatively small sample size and short follow‐up period. Second, the regimens, dose intensities, and durations of PC and AC were heterogeneous. In this study, some patients with pStage 0 or I were included. Patients with pStage 0 or I are not candidates for AC, however, the indication of AC for patients who have received PC is still unclear. Besides, some patients continued AC beyond a year post‐surgery, and doublet AC was administered to some patients with pStage II as well as patients with pStage III considering potential recurrence risks or patients' intentions, although it contradicts treatment guidelines recommendations. This heterogeneity may have introduced biases that affected patient survival outcomes following curative gastrectomy. Despite these limitations, this is, to our knowledge, the first study to examine the optimal timing for AC initiation in patients with GC who underwent PC followed by curative gastrectomy. These results provide valuable insights into the importance of early AC initiation within perioperative chemotherapy protocols for patients with advanced GC.

In conclusion, this study suggests that delaying AC initiation beyond 8 weeks worsens postoperative survival in patients with GC undergoing curative gastrectomy, regardless of PC status. To enhance patient outcomes, it is crucial to minimize postoperative complications and maintain patients' physical condition to enable a smooth transition to postoperative treatment.

## AUTHOR CONTRIBUTIONS

**Masataka Shimonosono:** Conceptualization; data curation; formal analysis; funding acquisition; investigation; methodology; project administration; writing – original draft. **Takaaki Arigami:** Conceptualization; data curation; methodology; supervision; validation; writing – review and editing. **Daisuke Matsushita:** Conceptualization; data curation; investigation; supervision; validation; writing – review and editing. **Yusuke Tsuruda:** Conceptualization; data curation; investigation; validation; writing – review and editing. **Ken Sasaki:** Conceptualization; data curation; supervision; writing – review and editing. **Kenji Baba:** Conceptualization; data curation; supervision; writing – review and editing. **Takao Ohtsuka:** Conceptualization; supervision; validation; writing – review and editing.

## FUNDING INFORMATION

This work was supported in part by a grant‐in‐aid (No. 24 K11892) for scientific research from Japan Society for the Promotion of Science.

## CONFLICT OF INTEREST STATEMENT

Takaaki Arigami has received lecture fees from Bristol Myers Squibb and Daiichi Sankyo, Japan. The other authors declare no conflicts of interest.

## ETHICS STATEMENT

Approval of the research protocol by an Institutional Reviewer Board: This study was approved by the Ethics Committee of the Kagoshima University (Approval number: 240052) and was conducted in accordance with the 1964 Declaration of Helsinki and later versions.

Informed Consent: This retrospective study used the “opt‐out” method to obtain informed consent from patients.

Registry and the Registration No. of the study/trial: N/A.

Animal Studies: N/A.

## Supporting information


**Table S1.** Regimens of preoperative chemotherapy.
**Table S2.** Regimens of adjuvant chemotherapy.
