# Supplementary material for: The impact of delayed adjuvant chemotherapy on survival in gastric cancer patients with and without preoperative chemotherapy
Source: Ann Gastroenterol Surg. 2025 Jan 16;9(4):668–77. doi: 10.1002/ags3.12911 (PMC12211110; doi:10.1002/ags3.12911)
Supplement: Supplementary file 1 — Table S1. Regimens of preoperative chemotherapy. Table S2. Regimens of adjuvant chemotherapy. [file AGS3-9-668-s001.pptx]

## Slide 1
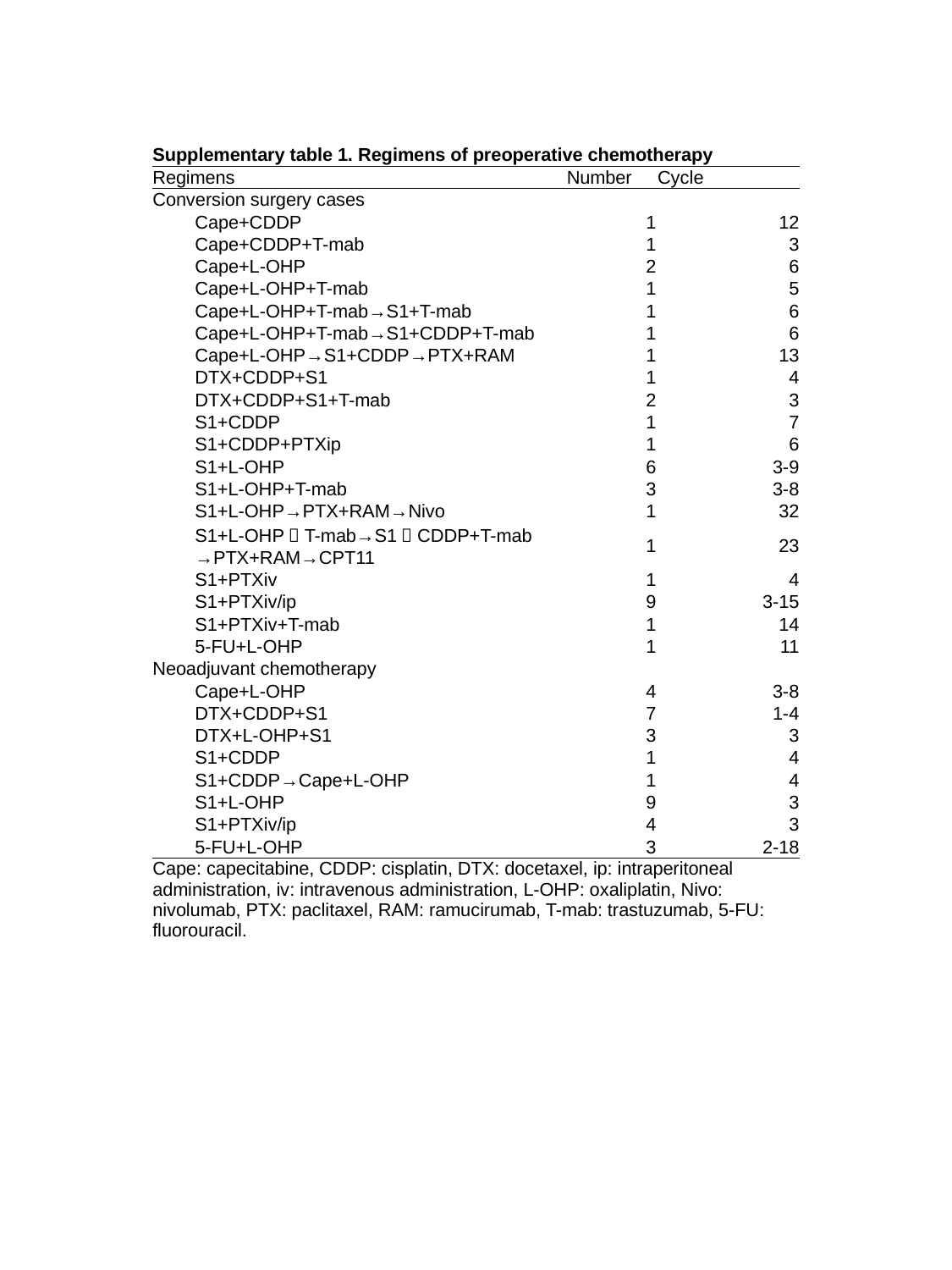

| Supplementary table 1. Regimens of preoperative chemotherapy | | | | |
| --- | --- | --- | --- | --- |
| Regimens | | | Number | Cycle |
| Conversion surgery cases | | | | |
| | Cape+CDDP | | 1 | 12 |
| | Cape+CDDP+T-mab | | 1 | 3 |
| | Cape+L-OHP | | 2 | 6 |
| | Cape+L-OHP+T-mab | | 1 | 5 |
| | Cape+L-OHP+T-mab→S1+T-mab | | 1 | 6 |
| | Cape+L-OHP+T-mab→S1+CDDP+T-mab | | 1 | 6 |
| | Cape+L-OHP→S1+CDDP→PTX+RAM | | 1 | 13 |
| | DTX+CDDP+S1 | | 1 | 4 |
| | DTX+CDDP+S1+T-mab | | 2 | 3 |
| | S1+CDDP | | 1 | 7 |
| | S1+CDDP+PTXip | | 1 | 6 |
| | S1+L-OHP | | 6 | 3-9 |
| | S1+L-OHP+T-mab | | 3 | 3-8 |
| | S1+L-OHP→PTX+RAM→Nivo | | 1 | 32 |
| | S1+L-OHP＋T-mab→S1＋CDDP+T-mab →PTX+RAM→CPT11 | | 1 | 23 |
| | S1+PTXiv | | 1 | 4 |
| | S1+PTXiv/ip | | 9 | 3-15 |
| | S1+PTXiv+T-mab | | 1 | 14 |
| | 5-FU+L-OHP | | 1 | 11 |
| Neoadjuvant chemotherapy | | | | |
| | Cape+L-OHP | | 4 | 3-8 |
| | DTX+CDDP+S1 | | 7 | 1-4 |
| | DTX+L-OHP+S1 | | 3 | 3 |
| | S1+CDDP | | 1 | 4 |
| | S1+CDDP→Cape+L-OHP | | 1 | 4 |
| | S1+L-OHP | | 9 | 3 |
| | S1+PTXiv/ip | | 4 | 3 |
| | 5-FU+L-OHP | | 3 | 2-18 |
| Cape: capecitabine, CDDP: cisplatin, DTX: docetaxel, ip: intraperitoneal administration, iv: intravenous administration, L-OHP: oxaliplatin, Nivo: nivolumab, PTX: paclitaxel, RAM: ramucirumab, T-mab: trastuzumab, 5-FU: fluorouracil. | | | | |

## Slide 2
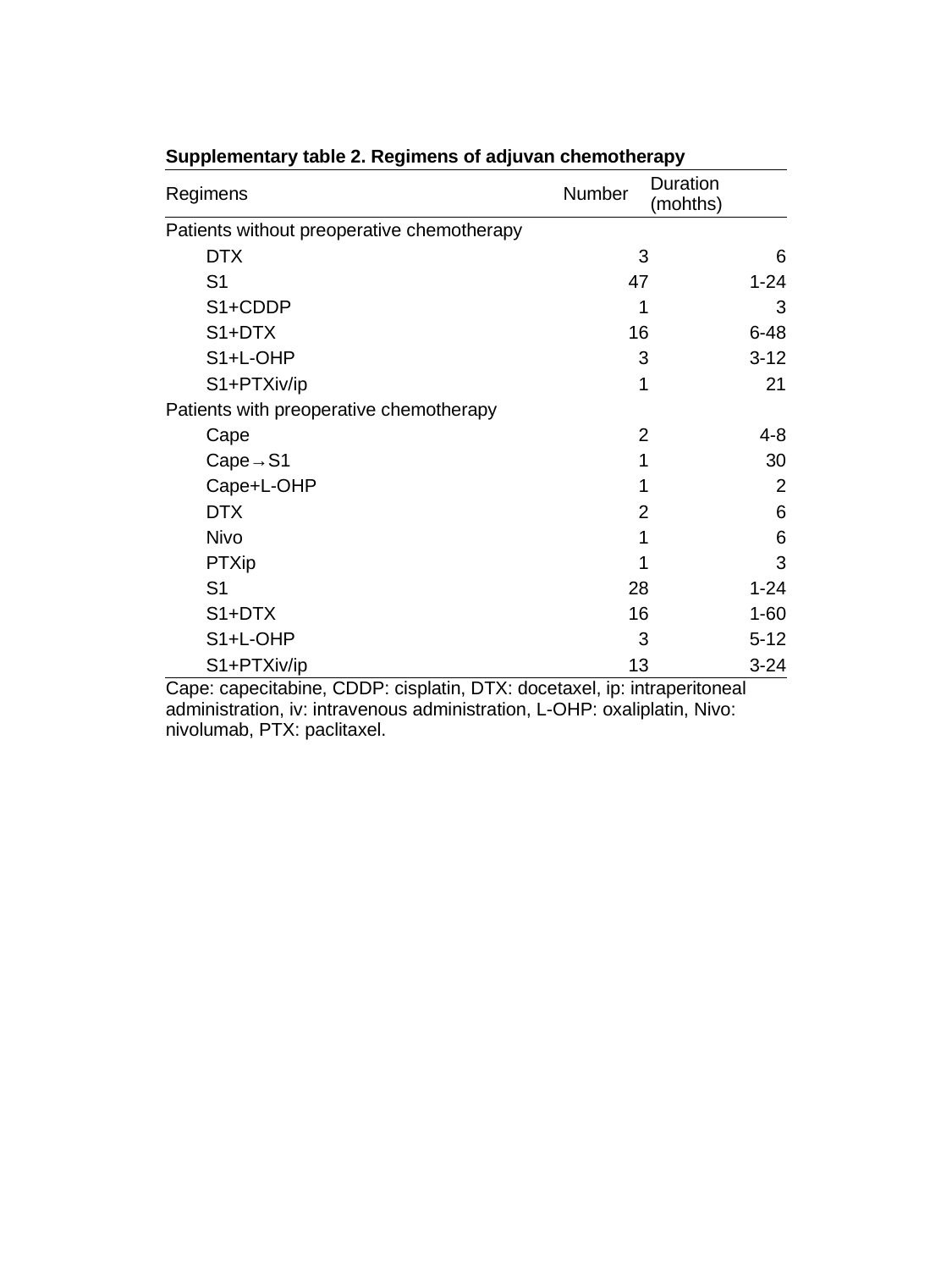

| Supplementary table 2. Regimens of adjuvan chemotherapy | | | | |
| --- | --- | --- | --- | --- |
| Regimens | | | Number | Duration (mohths) |
| Patients without preoperative chemotherapy | | | | |
| | DTX | | 3 | 6 |
| | S1 | | 47 | 1-24 |
| | S1+CDDP | | 1 | 3 |
| | S1+DTX | | 16 | 6-48 |
| | S1+L-OHP | | 3 | 3-12 |
| | S1+PTXiv/ip | | 1 | 21 |
| Patients with preoperative chemotherapy | | | | |
| | Cape | | 2 | 4-8 |
| | Cape→S1 | | 1 | 30 |
| | Cape+L-OHP | | 1 | 2 |
| | DTX | | 2 | 6 |
| | Nivo | | 1 | 6 |
| | PTXip | | 1 | 3 |
| | S1 | | 28 | 1-24 |
| | S1+DTX | | 16 | 1-60 |
| | S1+L-OHP | | 3 | 5-12 |
| | S1+PTXiv/ip | | 13 | 3-24 |
| Cape: capecitabine, CDDP: cisplatin, DTX: docetaxel, ip: intraperitoneal administration, iv: intravenous administration, L-OHP: oxaliplatin, Nivo: nivolumab, PTX: paclitaxel. | | | | |
